# Retraction: Silencing GADD153/CHOP Gene Expression Protects against Alzheimer’s Disease-Like Pathology Induced by 27-Hydroxycholesterol in Rabbit Hippocampus

**DOI:** 10.1371/journal.pone.0307751

**Published:** 2024-07-19

**Authors:** The PLOS ONE Editors

After this article [1] was published, concerns were raised about results presented in Figs 2, 3, 5, 6 and Figure S1. Specifically:

- The BACE1 panel in Fig 2C appears similar to the BACE1 panel in the siRNA duplex A section in Figure S1 and to the BACE1 panel in the siRNA duplex B section in Figure S1 despite representing different experiments.
- The β-actin panel in Fig 2C appears similar to the β-actin panel in Fig 3A.
- The β-actin panel in Fig 5A appears similar to the β-actin panel in Fig 6A.
- In the IRP-1 panel in Fig 6A, lanes 3 and 4 appear similar to each other, and there appears to be a vertical discontinuity in the background between lanes 3 and 4 suggestive of possible image splicing.

The authors did not respond in full to queries about the experiments in Figs 2, 3, 5, 6 and Figure S1.

In light of the extent and nature of the concerns listed above that question the reliability of the reported results and conclusions, the *PLOS ONE* Editors retract this article.

JRPP, TL and JS either did not respond directly or could not be reached. OG did not respond directly to the editorial decision.
